# Research on the anti-oxidant and anti-aging effects of *Polygonatum kingianum* saponins in *Caenorhabditis elegans*

**DOI:** 10.1016/j.heliyon.2024.e35556

**Published:** 2024-08-02

**Authors:** Yaqi Huang, Yetong Wang, Jia Deng, Sijie Gao, Jiakang Qiu, Jiawei He, Tong Yang, Nianhua Tan, Shaowu Cheng, Zhenyan Song

**Affiliations:** a School of Integrated Chinese and Western Medicine, Hunan University of Chinese Medicine, Changsha, Hunan, 410208, China; b Key Laboratory of Hunan Province for Integrated Traditional Chinese and Western Medicine on Prevention and Treatment of Cardio-Cerebral Diseases, Hunan University of Chinese Medicine, Changsha, Hunan, 410208, China

**Keywords:** *Polygonatum kingianum* saponins, *Caenorhabditis elegans*, Anti-aging, Anti-oxidant, SKN-1

## Abstract

Oxidative stress and its impact on aging are critical areas of research. Natural anti-oxidants, such as saponins found in *Polygonatum sibiricum*, hold promise as potential clinical interventions against aging. In this study, we utilized the nematode model organism, *Caenorhabditis elegans*, to investigate the pharmacological effects of *Polygonatum sibiricum* saponins (PKS) on antioxidation and anti-aging. The results demonstrated a significant anti-aging biological activity associated with PKS. Through experiments involving lifespan and stress, lipofuscin, q-PCR, and ROS measurement, we found that PKS effectively mitigated aging-related processes. Furthermore, the mechanism underlying these anti-aging effects was linked to the SKN-1 signaling pathway. PKS increased the nuclear localization of the SKN-1 transcription factor, leading to the up-regulation of downstream anti-oxidant genes, such as gst-4 and sod-3, and a substantial reduction in intracellular ROS levels within the nematode. In conclusion, our study sheds light on the anti-oxidant and anti-aging properties of PKS in *C. elegans*. This research not only contributes to understanding the biological mechanisms involved but also highlights the potential therapeutic applications of these natural compounds in combating aging-related processes.

## 1 Introduction

Aging is a degenerative physiological process characterized by a decline in biological functions and diminished adaptability and resistance. Oxidative stress damage is closely associated with aging [1,2]. Under normal conditions, the body maintains a dynamic balance between oxidation and anti-oxidation. However, with aging, this equilibrium is disrupted, leading to an excess of free radicals and/or a deficiency in anti-oxidants. This imbalance results in irreversible damage to biological membrane structures, disruption of organelle functions, and damage to macromolecules such as proteins and lipids [3]. In the aerobic metabolism of aging organisms, increased free radicals or anti-oxidant deficiency heightens cellular toxicity and reduces the body's anti-oxidant capacity. This shift induces irreversible damage to the structures of biological membranes, amino acid chains, and DNA molecules, ultimately accelerating the aging process and the onset of age-related degenerative diseases [4].

*C. elegans*, due to its simple structure, short lifespan, ease of manipulation, and convenient observation, serves as an ideal model organism in aging research. It allows for the rapid assessment of potential anti-oxidant activity of preclinical drugs. Approximately 60%–80 % of *C. elegans* genes are highly conserved with human genes, including the homologous gene of nuclear factor E2-related factor 2(Nrf2), known as Protein skinhead-1(SKN-1) [5].

Nrf2 is a crucial transcription factor regulating anti-oxidant stress responses, with Kelch-like ECH-associated protein 1 (Keap1) serving as its specific receptor. Under physiological conditions, Nrf2 primarily exists in the cytoplasm, forming a complex with Keap1 to inhibit Nrf2 activity. However, under oxidative stress, Nrf2 dissociates from Keap1, undergoes nuclear translocation, binds with anti-oxidant response elements (ARE), and initiates phase II detoxifying enzyme and anti-oxidant enzyme gene expression. This process enhances cellular resistance to oxidative stress and nucleophilic compounds [6]. Therefore, the Keap1-Nrf2/ARE signaling pathway is a primary induced defense pathway in the body against oxidative and electrophilic stress [7], playing a crucial role in the body's response to oxidative stress and harmful stimuli. It is considered the most important endogenous anti-oxidant signaling pathway in the body.

With the establishment of aging models and aging phenotype indicators, various natural plant extracts have been shown to prevent and/or delay aging, holding promise as adjuncts for longevity. *Polygonatum sibiricum* Delar. ex Redoute, commonly known as Huang Jing, is a perennial herbaceous plant of the Liliaceae family, and a traditional Chinese medicine (TCM). According to TCM, Huang Jing is believed to possess various effects, including tonifying the spleen and nourishing the lungs, invigorating the middle, benefiting Qi, beautifying the skin, nourishing the kidneys and essence, and strengthening bones and marrow [8,9]. Modern pharmacological research indicates that Huang Jing has various pharmacological effects, including immunomodulation, anti-oxidant activity, anti-aging properties, cardiovascular system protection, liver and bone health, regulation of blood lipids, and anti-tumor activity [10]. Huang Jing has a highly diverse composition of bioactive compounds such as saponins, polysaccharides, flavones, alkaloids, lignins, etc. Because of such diverse composition, Huang Jing has been used as an anti-aging, anti-inflammatory, anti-osteoporotic agent, as well as an immunity booster, sleep enhancer, etc [11]. Studies have shown that *Polygonatum kingianum* polysaccharides (PSP) can alleviate oxidative stress and improve renal function in aging rats induced by d-galactose by acting on Klotho-FGF23 endocrine axis and up-regulating Klotho mRNA and K1otho protein expression in renal cortex, thus playing an important role in delaying aging [12,13]. However, previous studies have predominantly focused on polygahatous polysaccharides, with limited reports on the relationship between *Polygonatum kingianum* saponins (PKS) and anti-aging effects [14,15].

In recent studies, including the work by Yang et al. [16], PKS has been shown to delay cellular senescence and extend the lifespan of Caenorhabditis elegans through various mechanisms. It demonstrated that PKS exerts its anti-aging effects via down-regulation of the senescence-associated secretory phenotype (SASP) and activation of the sir-2.1/autophagy pathway. Our research complements these findings by exploring the anti-oxidative properties of PKS, specifically through the SKN-1/NRF2 signaling pathway, thereby providing a broader understanding of the anti-aging mechanisms of PKS. This research enriches our understanding of the medicinal value of Huang Jing, providing new insights for clinical anti-aging studies and the development of TCM.

## 2 Materials and methods

### 2.1 Extraction and identification of *Polygonatum kingianum* saponins (PKS)

Following the methodologies outlined by He S [17], 120 g of Polygonatum sibiricum powder was ground, subjected to petroleum ether ultrasound, filtered, and the residue was subjected to ethanol ultrasound followed by filtration. After dissolving in hydrochloric acid and placement in a separatory funnel, extraction with n-butanol was performed thrice. The combined n-butanol extracts were evaporated, freeze-dried, yielding the crude extract of PKS. The extract was diluted to 0.57 mg/mL, passed through an AB-8 macroporous resin column, and eluted with ethanol. The eluate was collected, evaporated, and freeze-dried. The concentration of PKS was determined using a standard curve generated with sarsasapogenin standard, measured at the maximum absorption wavelength of 560 nm.

### 2.2 Cultivation conditions for *C. elegans* and Escherichia coli OP50

Unless otherwise specified, *C. elegans* strains, including wild-type N2, CB1370[daf-2(e1370)], DA116[eat-2(1116 CE)], EU1[skn-1(zu67)], and LD1 (ldIs7 [skn-1b/cGFP + rol-6(su1006)]) were maintained on nematode growth medium (NGM) plates containing Escherichia coli OP50 bacteria obtained from the Caenorhabditis Genetics Center (CGC, University of Minnesota, Minneapolis, MN, USA) at 20 °C. Synchronized populations were obtained by sodium hypochlorite treatment of hermaphrodites [18].

### 2.3 Toxicity assays

Synchronized L4 stage N2 wild-type *C. elegans* were transferred to culture dishes containing different concentrations of PKS (50, 100, 200, 400, 800, 1600, 3200 μg/mL) and blank dishes with bacteria. After 48 and 72 h of cultivation, worm survival was observed. *C. elegans* were considered dead if they did not respond to gentle platinum wire prodding after 10 s or if they were excluded due to accidental death, escape (climbing and drying on the wall), bagging, or internal hatching [19].

### 2.4 Lifespan assays

L4 stage N2 *C. elegans* were transferred to plates containing PKS (50, 100, 200 μg/mL) and blank plates with bacteria, with 50 *C. elegans* per group. *C. elegans* were transferred to new plates daily to ensure drug efficacy and were gently prodded with a platinum wire, recording the number of surviving *C. elegans* daily until the last worm died [20].

### 2.5 Stress resistance assays

To assess resistance to paraquat, synchronized L4 stage N2 *C. elegans* were treated with PKS (50, 100, 200 μg/mL) for 3 days. Afterward, they were transferred to plates containing 10 mmol·L-1 paraquat for oxidative stress treatment. Survival was recorded every 12 h until the last worm died. Resistance to ultraviolet (UV) and high temperature was evaluated by transferring *C. elegans* to NGM plates without OP50. UV stress involved exposure to open UV light for 2 h in a laminar flow hood, while heat stress included placing the *C. elegans* at 35 °C for 2 h. *C. elegans* were then transferred to plates with the corresponding concentrations of bacteria and survival was recorded daily until the last worm died [21].

### 2.6 Lipofuscin analysis

L4 stage N2 *C. elegans* were treated with PKS (50, 100, 200 μg/mL), and after 7 days, a subset of *C. elegans* from the 200 μg/mL PKS and blank groups were transferred to plates containing 10 mM paraquat. After 12 h, *C. elegans* were picked onto glass slides with 2 % agarose gel and anesthetized with 10 μL of a 10 mmol·L-1 levamisole solution. Fluorescence intensity was observed under an inverted fluorescence microscope, and Image-Pro Plus software was used for quantitative analysis of fluorescence intensity [22].

### 2.7 Physiological function experiments

Swallowing frequency and body swing frequency were determined on days 3, 5, 7, and 9 after treatment. *C. elegans* was transferred to NGM plates without OP50, and their responses were recorded after 1 min of acclimatization. For the reproductive assay, five *C. elegans* from each group were transferred to new plates each day and allowed to lay eggs for four consecutive days. The number of offspring was counted 2 days after laying [23].

### 2.8 Stress and lifespan experiments in mutant *C. elegans*

L4 stage daf-2, eat-2, skn-1 mutant *C. elegans*, and N2 wild-type *C. elegans* were transferred to plates containing 200 μg/mL PKS and blank plates with bacteria (50 *C. elegans* per group). Oxidative stress assays followed the methodology described in section 2.5 [23]. Lifespan experiments for skn-1 mutant *C. elegans* were conducted using the same methodology as described in section 2.4.

### 2.9 SKN-1 Green Fluorescent protein (GFP) cellular localization assay

L4 stage SKN-1(B/C)GFP *C. elegans* were treated with PKS (200 μg/mL) for 7 days, and a subset of *C. elegans* from the 200 μg/mL PKS and blank groups were transferred to plates containing 10 mmol·L-1 paraquat for 12 h. Afterward, *C. elegans* were picked onto glass slides with 2 % agarose gel and anesthetized with 10 μL of a 10 mmol·L-1 levamisole solution. SKN-1(GFP) localization was observed under an inverted fluorescence microscope, and Image-Pro Plus software was used for quantitative analysis [24].

### 2.10 q-PCR experiments

The L4 stage N2 *C. elegans* were grouped and treated as in Section 2.6, and RNA was extracted from 100 *C. elegans* per group following the manufacturer's instructions. Reverse transcription was performed, and Quantitative Real-time PCR(q-PCR) using SYBR Green dye was conducted to assess the expression of gst-4, gst-7, sod-3, and hsp16.2 genes with GAPDH as the reference gene. The same methodology was applied for skn-1 mutant *C. elegans* treated with PKS, as described in section 2.9. The primer sequence is shown in Table 1.

**Table 1:** Primer sequences.

Primer	Forward primer(5’→3′)	Reverse primer(3’→5′)	Length/bp
GAPDH	GGAAGTCGCAGCACAAGAT	AGCAGATGGAGCAGAGATGAT	176
gst-4	TGATGCTCGTGCTCTTGCT	TGATGCTCGTGCTCTTGCT	167
gst-7	CGGATACTTGGTTGGAGACTCT	CGGATACTTGGTTGGAGACTCT	156
sod-3	TTGGCTAAGGATGGTGGAGAA	GAACCGCAATAGTGATGTCAGA	112
hsp16.2	CGCTATCAATCCAAGGAGAACA	GCAACTGCACCAACATCTACA	109

### 2.11 ROS level detection experiment

The L4 stage N2 *C. elegans* were grouped and treated as in Section 2.6, and ROS levels were measured using the H2DCF-DA fluorescence probe. The fluorescence intensity was recorded every 5 min for 60 min in a microplate reader, and the concentration of ROS was determined. The same methodology was applied for skn-1 mutant *C. elegans* treated with PKS, as described in section 2.9.

### 2.12 Statistical analysis

Statistical analyses were performed using Prism GraphPad 8.3 software. Survival curve results were analyzed using the Log-Rank test, with *P* < 0.05 considered statistically significant. Other experiments were compared between two groups using analysis of variance (ANOVA), with *P* < 0.05 considered statistically significant.

## 3 Results

### 3.1 Identification of total saponin content and toxicity detection of *Polygonatum kingianum* saponins (PKS)

**Supplementary Data Fig. 1:**
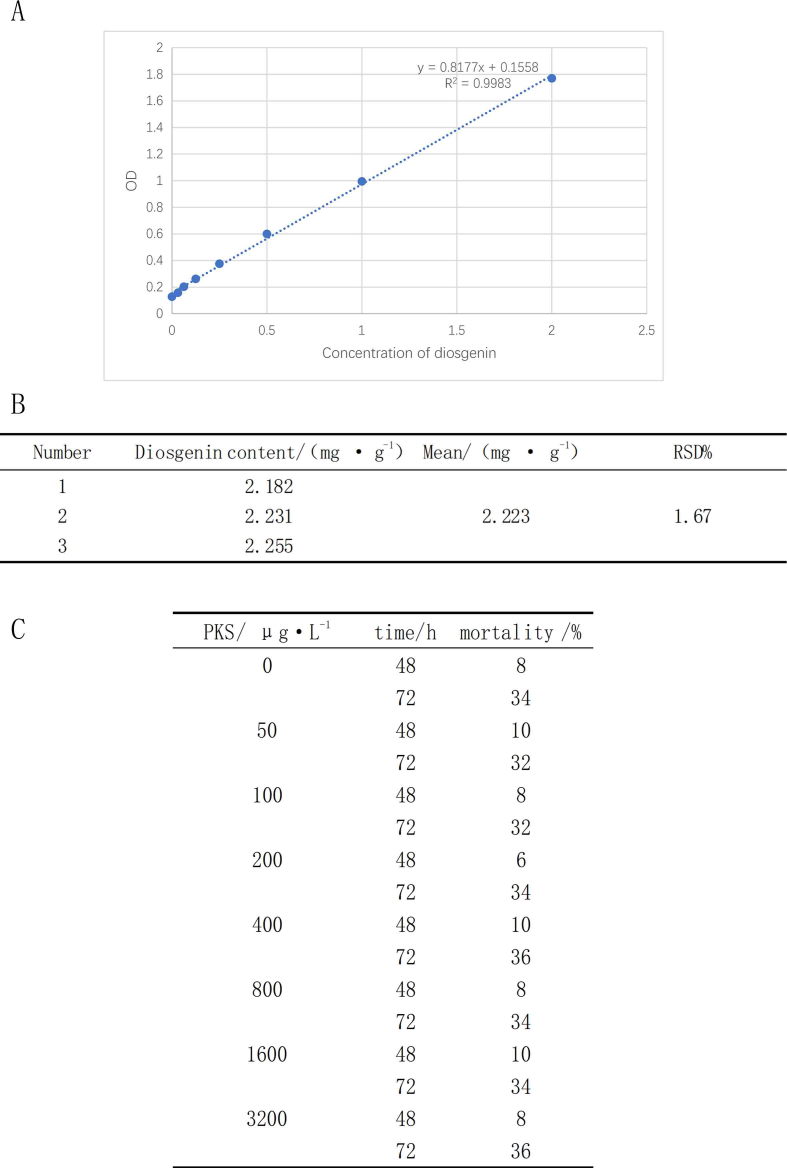
Standard curve equation of diosgenin saponins.

The linear equation of the Diosgenin standard curve was y = 0.8177x+0.1558 (r = 0.9983) (Supplementary Data Fig. 1). The mass concentration of saponins within the range of 0.0052–0.33 mg/mL exhibited a good linear relationship with absorbance. The calculated total saponin content in the extract was 2.223 mg/g (Table 2). Toxicity experiments on *Caenorhabditis elegans* (*C. elegans*) exposed to PKS indicated no toxic side effects at concentrations below 3.2 mg/mL. Therefore, subsequent experiments were conducted with 50, 100, and 200 μg/mL of PKS (Table 3).

**Fig. 1:**
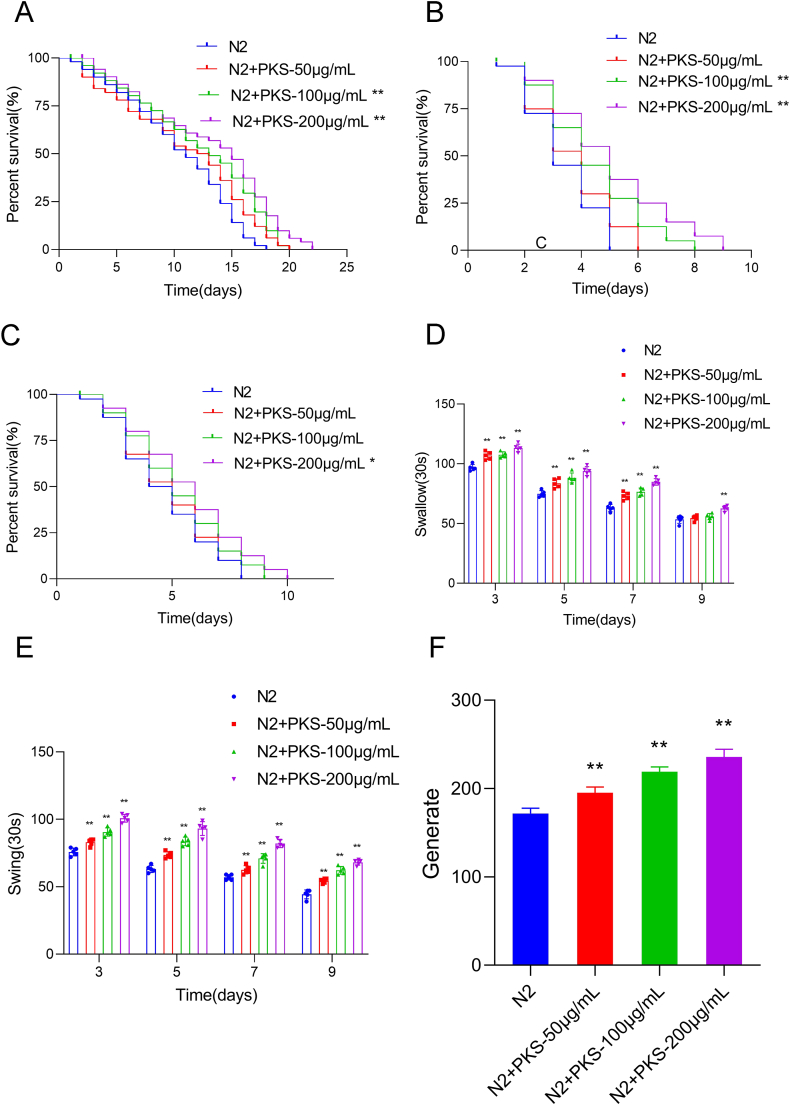
PKS extends the lifespan and enhances anti-aging capacity in *C. elegans*. (A) Influence of PKS on the lifespan of *C. elegans*. (B) Impact of PKS on *C. elegans* survival time under UV stress conditions. (C) Effect of PKS on *C. elegans* survival time under heat shock conditions. (D) Statistical analysis of swallowing frequency in *C. elegans* on days 3, 5, 7, and 9 treated with PKS. (E) Statistical analysis of body bending frequency in *C. elegans* on days 3, 5, 7, and 9 treated with PKS. (F) Impact of PKS on reproductive capacity in *C. elegans*. *P < 0.05, **P < 0.01 compared to N2 group. N = 50.

**Table 2:** Total saponin content in *Polygonatum Kingianum* Extracts.

Number	Diosgenin content/(mg/g)	Mean/(mg/g)	RSD%
1	2.182	2.223	1.67
2	2.231		
3	2.255

**Table 3:** Toxic effects of *Polygonatum Kingianum* Saponins on *C. elegans*.

PKS/μg·L^−1^	time/h	mortality/%
0	48	8
	72	34
50	48	10
	72	32
100	48	8
	72	32
200	48	6
	72	34
400	48	10
	72	36
800	48	8
	72	34
1600	48	10
	72	34
3200	48	8
	72	36

### 3.2 PKS prolongs the lifespan of *C. elegans* and enhances anti-aging capability

Survival analysis, a direct indicator of *C. elegans* aging, demonstrated that under normal laboratory conditions, the average lifespan of N2 *C. elegans* was 10.480 ± 0.655 days. Upon treatment with 50, 100, and 200 μg/mL of PKS, the average lifespan increased to 11.020 ± 0.796 days, 12.216 ± 0.755 days, and 13.137 ± 0.801 days, respectively (Fig. 1A). Compared to the N2 group, the lifespan of the 50, 100, and 200 μg/mL PKS groups increased by 5.15 %, 16.56 % (*P* < 0.01), and 25.35 % (*P* < 0.001), respectively, indicating a dose-dependent effect of PKS on extending the lifespan of adult *C. elegans*.

Lifespan extension is positively correlated with survival under various stressors [25]. Under ultraviolet (UV) stress conditions (Fig. 1B), 100 and 200 μg/mL of PKS significantly prolonged the average survival time to 4.425 ± 0.268 days (*P* < 0.01) and 5.025 ± 0.327 days (P < 0.001), respectively, compared to the N2 group. In the 35 °C heat shock condition (Fig. 1C), the average survival time of the N2 group was 4.650 ± 0.307 days, while 200 μg/mL of PKS extended the survival time to 5.700 ± 0.355 days (*P* < 0.05), indicating that PKS enhances resistance to UV and heat stress.

PKS significantly increased the swallowing frequency of wild-type N2 adult *C. elegans* on days 3, 5, 7, and 9 after treatment (*P* < 0.01) (Fig. 1D). Additionally, the bending frequency of *C. elegans* in M9 liquid, a widely used indicator of aging in *C. elegans*, was higher on days 3, 5, 7, and 9 after PKS treatment (*P* < 0.01) (Fig. 1E). Furthermore, PKS increased the number of offspring, a crucial indicator of physiological function, at all concentrations tested (*P* < 0.01) (Fig. 1F). Collectively, these results suggest that PKS delays the aging process of wild-type N2 *C. elegans* and enhances anti-aging capabilities. Notably, a concentration of 200 μg/mL PKS demonstrated the most pronounced intervention effect, thus being selected as the optimal dosage for investigating its anti-aging and anti-oxidant mechanisms.

### 3.3 PKS reduces lipofuscin and ROS levels in *C. elegans*, promoting the expression of anti-oxidant genes

**Fig. 2:**
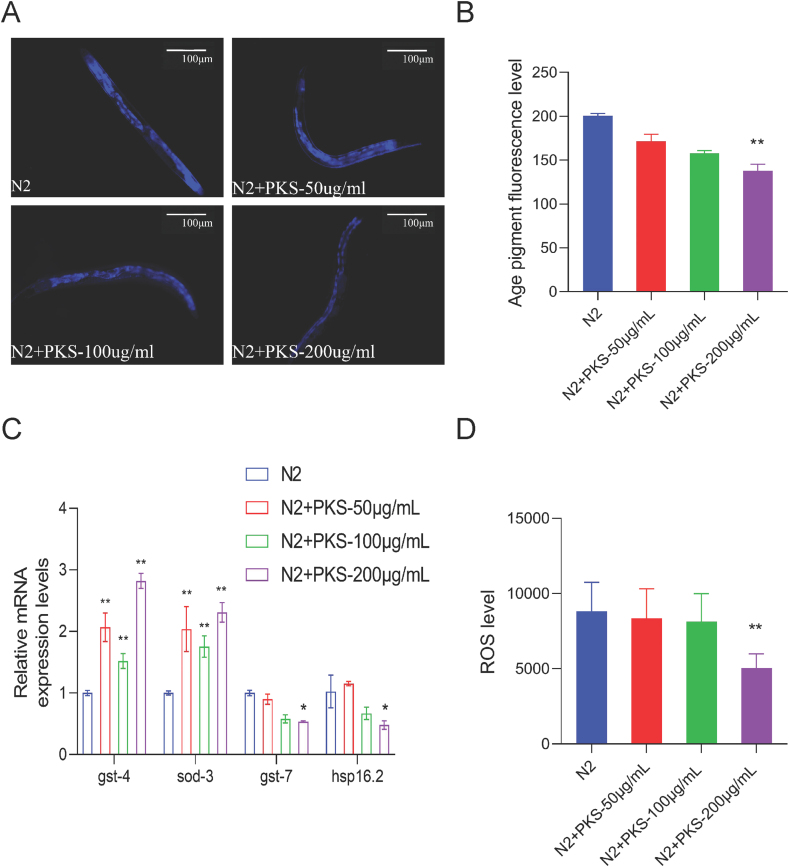
PKS reduces lipofuscin and ROS, inducing anti-oxidant gene expression in *C. elegans*. (A) Fluorescence images of lipofuscin under physiological conditions. (B) Statistical analysis of lipofuscin fluorescence intensity under physiological conditions. (C) qPCR detection of anti-oxidant gene expression (gst-4, gst-7, sod-3, and hsp-16.2) under physiological conditions. (D) Expression levels of endogenous ROS. *P < 0.05, **P < 0.01 compared to N2 group. N = 50.

Lipofuscin, an oxidative protein, accumulates in aging cells, leading to imbalances in cellular functions and serving as a crucial indicator of aging [26,27]. Compared to the N2 group, treatment with 200 μg/mL of PKS significantly reduced lipofuscin fluorescence intensity in *C. elegans* (*P* < 0.001) (Fig. 2A–B), indicating that PKS alleviates age-related physical decline.

ROS is a critical factor in oxidative damage, participating in the aging process [28]. The effect of PKS on endogenous ROS levels in N2 *C. elegans* was measured using the 2′,7′-Dichlorofluorescin diacetate (H2DCF-DA) fluorescence probe. Compared to the N2 group, treatment with 200 μg/mL of PKS significantly reduced endogenous ROS levels (*P* < 0.001) (Fig. 2D), suggesting that the lower ROS levels are associated with the extended healthspan and enhanced anti-oxidant stress capacity.

To investigate the direct factors contributing to the reduction in ROS induced by PKS, the mRNA expression of anti-oxidant genes gst-4, gst-7, sod-3, and hsp16.2 in N2 *C. elegans* was analyzed. Results revealed a significant upregulation in the expression levels of gst-4 and sod-3 after intervention with 200 μg/mL of PKS (*P* < 0.001) (Fig. 2C). The transcriptional activation of gst-4 is commonly used as a readout for SKN-1 activity [29,30].

### 3.4 PKS induces anti-oxidant stress, reducing damage in *C. elegans* under oxidative stress

**Fig. 3:**
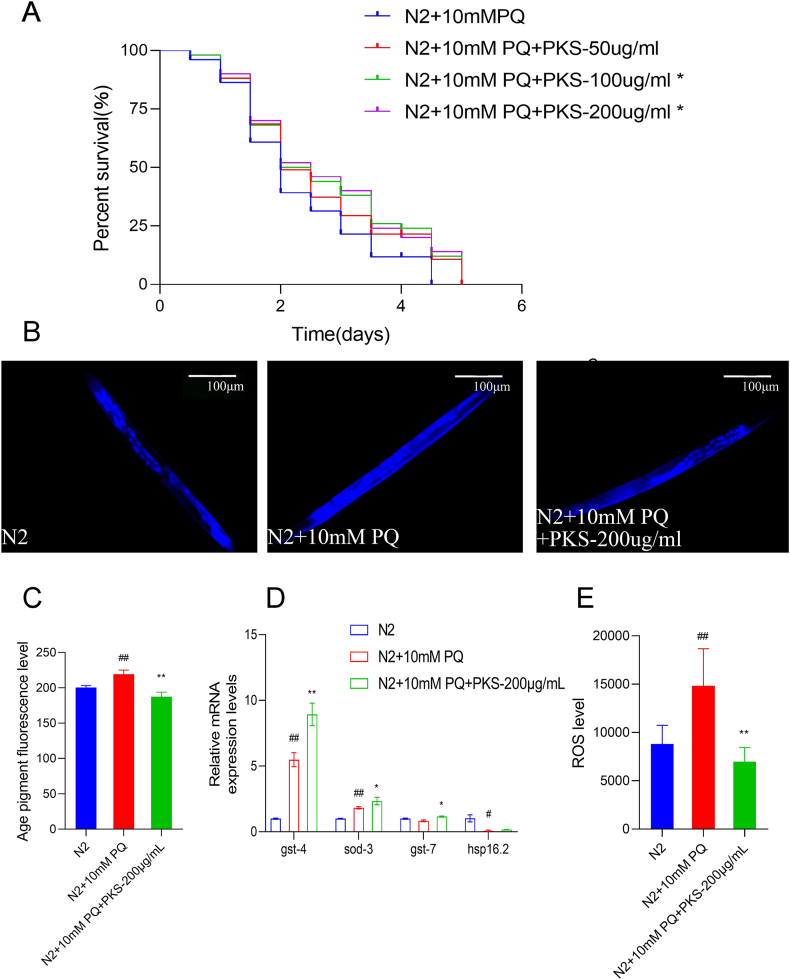
PKS induces anti-oxidant stress resistance and reduces damage in *C. elegans*. (A) Influence of PKS on worm lifespan under oxidative stress conditions. (B) Fluorescence images of lipofuscin under oxidative stress conditions. (C) Statistical analysis of lipofuscin fluorescence intensity under oxidative stress conditions. (D) qPCR detection of anti-oxidant gene expression (gst-4, gst-7, sod-3, and hsp-16.2) under oxidative stress conditions. (E) Expression levels of endogenous ROS under oxidative stress conditions. #*P* < 0.05, ##*P* < 0.01; *P < 0.05, **P < 0.01 compared to N2 and model groups, respectively. N = 40.

The utilization of PQ (paraquat) allows for the creation of an oxidative damage model. This model induces *Caenorhabditis elegans* to generate a substantial amount of reactive oxygen species (ROS). The excessive accumulation of ROS leads to mitochondrial dysfunction, thereby expediting the aging process [31]. The study found that 100 and 200 μg/mL of PKS significantly prolonged the lifespan of *C. elegans* under oxidative stress conditions (*P* < 0.05) (Fig. 3A). Moreover, under oxidative stress conditions, 200 μg/mL of PKS significantly reduced lipofuscin fluorescence intensity (*P* < 0.001) (Fig. 3B–C) and increased the expression levels of anti-oxidant-related genes gst-4 and sod-3 (P < 0.001, *P* < 0.05) (Fig. 3D). The reduction in ROS levels in N2 *C. elegans* treated with 200 μg/mL of PKS under oxidative stress was also significant (*P* < 0.001) (Fig. 3E). Overall, PKS extends the lifespan of *C. elegans* under oxidative stress, exhibits anti-oxidant activity, and reduces ROS production under oxidative stress conditions, possibly through the regulation of anti-oxidant genes.

### 3.5 PKS induces oxidative stress resistance via the SKN-1 pathway

**Fig. 4:**
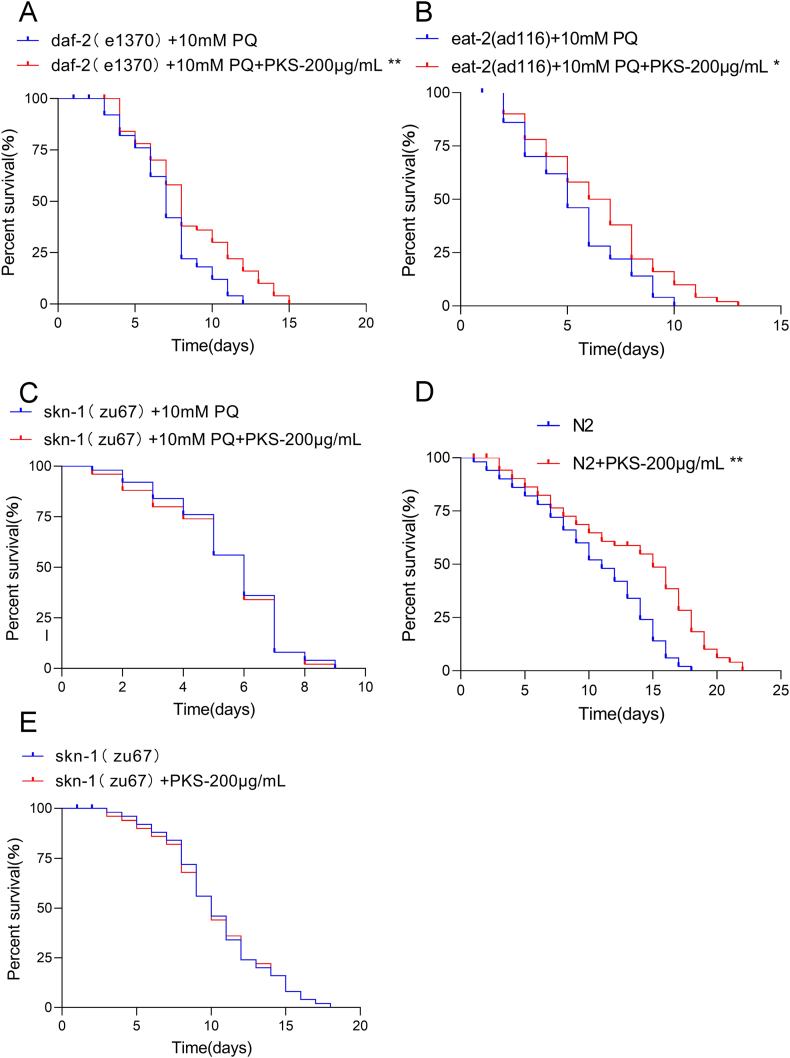
PKS induces oxidative stress resistance via the SKN-1 pathway. (A) Survival curves of daf-2 mutant *C. elegans* under oxidative stress conditions. (B) Survival curves of eat-2 mutant *C. elegans* under oxidative stress conditions. (C) Survival curves of skn-1 mutant *C. elegans* under oxidative stress conditions. (D) Survival curves of N2 *C. elegans* under physiological conditions. (E) Survival curves of skn-1 mutant *C. elegans* under physiological conditions. *P < 0.05, **P < 0.01 compared to respective control groups. N = 40.

Utilizing daf-2 mutant *C. elegans* associated with the aging-related insulin signaling pathway, eat-2 mutant *C. elegans* associated with dietary restriction signaling, and skn-1 mutant *C. elegans* with the transcription factor SKN-1, the study explored the anti-oxidative action pathway of PKS in vivo [23]. Results showed that 200 μg/mL of PKS extended the lifespan of daf-2 (Fig. 4A) and eat-2 (Fig. 4B) mutant *C. elegans* under oxidative stress (*P* < 0.01, *P* < 0.05), but had no effect on the lifespan of skn-1 mutant *C. elegans* (*P* > 0.05) (Fig. 4C). In normal physiological conditions, 200 μg/mL of PKS extended the lifespan of N2 *C. elegans* (Fig. 4D), but not that of skn-1 mutant *C. elegans* (Fig. 4E), suggesting that the lifespan extension induced by PKS is related to the regulation of the SKN-1 signaling pathway, rather than the DAF-2 and EAT-2 pathways.

### 3.6 PKS induces oxidative stress resistance in *C. elegans* requires SKN-1

**Fig. 5:**
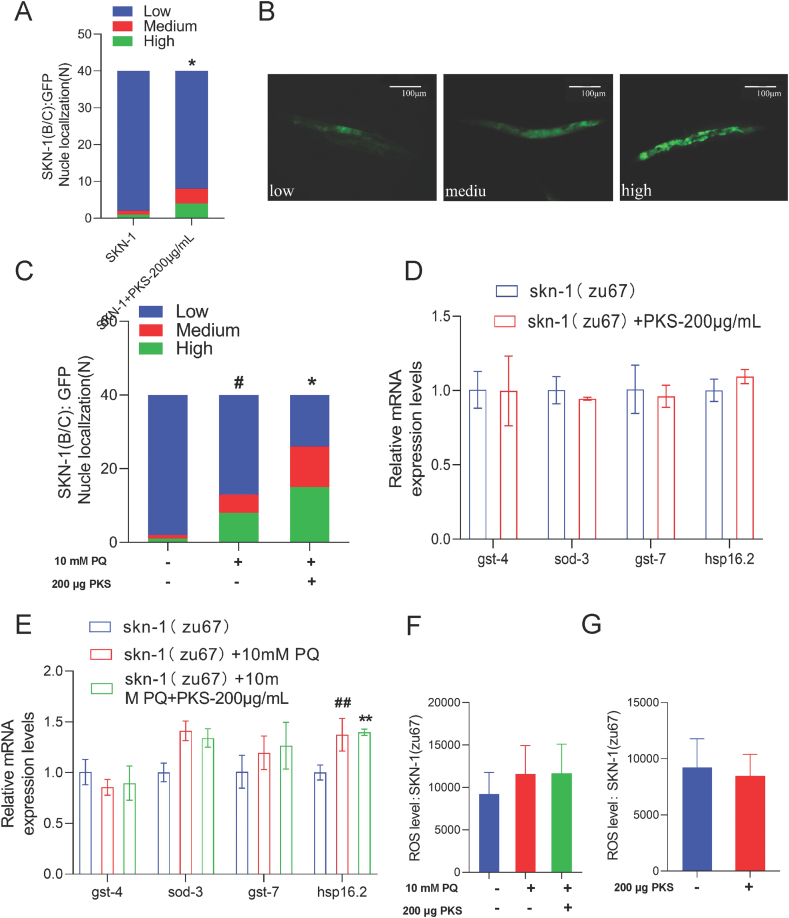
PKS induces oxidative stress resistance in *C. elegans* via the SKN-1 pathway. (A) Influence of PKS on SKN-1(B/C)GFP expression under physiological conditions. (B) Image of SKN-1 nuclear translocation in SKN-1(B/C)GFP *C. elegans*. (C) Influence of PKS on SKN-1(B/C)GFP expression under oxidative stress conditions. (D) qPCR detection of anti-oxidant gene expression (gst-4, gst-7, sod-3, and hsp-16.2) in skn-1 mutant *C. elegans* under physiological conditions. (E) qPCR detection of anti-oxidant gene expression (gst-4, gst-7, sod-3, and hsp-16.2) in skn-1 mutant *C. elegans* under oxidative stress conditions. (F) Expression levels of endogenous ROS in skn-1 mutant *C. elegans* under oxidative stress conditions. (G) Expression levels of endogenous ROS in skn-1 mutant *C. elegans* under physiological conditions. *P < 0.05 compared to SKN-1(GFP) group; #*P* < 0.05 compared to the model group; *P < 0.05 compared to respective control groups. N = 40.

SKN-1, the homolog of the mammalian anti-oxidant regulator NRF2 in *C. elegans*, promotes oxidative stress resistance and helps extend lifespan [32]. To further assess SKN-1 activity, the nuclear localization of SKN-1 in SKN-1(B/C)GFP *C. elegans* was examined, revealing a significant promotion of SKN-1 nuclear entry after treatment with 200 μg/mL of PKS (*P* < 0.05) (Fig. 5A–B). Under oxidative stress conditions, 200 μg/mL of PKS also promoted SKN-1 nuclear entry (*P* < 0.05) (Fig. 5C), indicating that SKN-1 nuclear entry is activated to exert downstream transcriptional effects in response to oxidative stress.

The SKN-1/NRF2 receptor signaling pathway plays a crucial role in the aging process of *C. elegans* [33]. To determine if PKS acts through this mechanism, the mRNA expression of gst-4, gst-7, sod-3, and hsp16.2 was analyzed in skn-1 mutant *C. elegans*. Results showed that in skn-1 mutant *C. elegans*, there was no significant difference in the expression levels of downstream anti-oxidant genes gst-4, sod-3, gst-7, and hsp16.2 after intervention with 200 μg/mL of PKS (*P* > 0.05) (Fig. 5D). Similarly, under oxidative stress conditions, PKS had no impact on the expression of these genes (*P* > 0.05) (Fig. 5E). This suggests that the antioxidative activity of PKS is likely associated with the regulation of SKN-1 transcription factor and its downstream anti-oxidant genes gst-4 and sod-3, further indicating that PKS extends the lifespan of *C. elegans* by regulating the downstream anti-oxidant genes gst-4 and sod-3 through SKN-1 transcription factor.

To investigate the potential relationship between SKN-1/NRF2 receptor signaling and the reduction of ROS, studies utilizing skn-1 mutant nematodes revealed no significant change (*P* > 0.05) in endogenous ROS levels following treatment with 200 μg/mL PKS (Fig. 5F). However, when exposed to oxidative stress conditions, the endogenous ROS levels in SKN-1 mutant nematodes remained non-significant (*P* > 0.05) even after treatment with 200 μg/mL PKS (Fig. 5G), suggesting that the regulation of ROS production in nematodes may be influenced by the transcription factor SKN-1.In conclusion, PKS may potentially extend the lifespan of *C. elegans* by modulating the SKN-1 transcription factor to augment anti-oxidant capacity and thereby mitigate endogenous ROS production.

## 4 Discussion

*Polygonatum sibiricum* Delar. ex Redoute (Huangjing, Chinese name), a traditional functional food with a history of several centuries, is renowned for its diverse pharmacological effects, including anti-aging and health promotion. Polysaccharides and saponins in Huangjing were traditionally considered as the most prominent bioactive constituents. Recent studies have highlighted the potential of Polygonatum kingianum saponins (PKS) in delaying cellular senescence and prolonging the healthy lifespan of *C. elegans*. For instance, Jing-Juan Yang et al. [16] reported that PKS delays cellular senescence by down-regulating the senescence-associated secretory phenotype (SASP) and activates the sir-2.1/autophagy pathway to extend the lifespan of *C. elegans*. Our findings corroborate and extend these results by demonstrating that PKS not only increases the lifespan but also enhances stress resistance in *C. elegans* via the SKN-1 signaling pathway. We observed significant reductions in lipofuscin and reactive oxygen species (ROS) levels, along with increased expression of anti-oxidant genes such as *gst-4* and *sod-3*. These results suggest that the anti-oxidant and anti-aging effects of PKS are mediated through the activation of SKN-1, a homolog of the mammalian Nrf2, which regulates oxidative stress responses. Furthermore, Our research emphasizes the role of SKN-1 in mediating the antioxidative effects of PKS and demonstrates the potential of PKS to modulate multiple pathways involved in aging and stress resistance. By elucidating these additional pathways, our study contributes to a more comprehensive understanding of how PKS can be utilized for anti-aging interventions.

The aging process in *C. elegans* is closely associated with oxidative stress [34], where the accumulation of oxidative damage resulting from an imbalance in reactive oxygen species (ROS) levels is considered a major cause of aging. Treatment with 10 mM paraquat results in the production of a large amount of ROS and thus has been widely used as a stressor in the mitochondrial respiratory chain [35]. In this experiment, treatment with PKS significantly prolonged the survival time of *C. elegans* under the conditions of using paraquat as oxidant, markedly reduced lipofuscin expression levels, and significantly decreased endogenous ROS levels in *C. elegans*, indicating that PKS may regulate the anti-aging response by reducing ROS in *C. elegans* under oxidative stress.

It has been reported that cellular defense responses to oxidative stress are regulated by many transcription factors, including SKN-1 and DAF-16 [36,37]. SKN-1 functions in the p38 MAPK pathway to regulate the oxidative stress response and in parallel to DAF-16/FOXO in the daf-2 mediated insulin/IGF-1-like signaling pathway to regulate lifespan [38]. Studies have shown that *C. elegans* can improve health and prolong life through the dietary restriction signaling (eat-2) [39]. Notably, PKS extended the lifespan of daf-2 and eat-2 mutant *C. elegans* under oxidative stress conditions, while the lifespan of skn-1 mutant *C. elegans* remained unaltered. These results indicate that the anti-oxidant activity of PKS is independent of insulin signaling (daf-2) and dietary restriction signaling (eat-2).

In *C. elegans*, the antioxidative activity of PKS is associated with the SKN-1/NRF-2 pathway. Previous studies have shown that Zhuyeqing liquor enhances resistance to adversity and promotes lifespan by modulating the SKN-1 and HSF-1 transcription factors in *C. elegans* [40]. Similarly, tauroursodeoxycholic acid promotes longevity and stress resistance through stress response factors DAF-16/FOXO and SKN-1/NRF2 in *C. elegans* [41]. Our results indicate that PKS induces translocation of SKN-1 from the cytoplasm to the nucleus under physiological and oxidative stress conditions, activating the downstream expression of anti-oxidant genes gst-4 and sod-3, which may be associated with lifespan extension. ROS is mainly cleared by anti-oxidant enzymes, among which the most important are sod-3 and gst-4 [42]. However, PKS fails to promote downstream gst-4 and sod-3 expression in skn-1 mutant *C. elegans* under physiological and oxidative stress conditions, and does not reduce ROS levels, further confirming that the anti-aging mechanism of PKS is related to the regulation of the classical SKN-1/NRF-2 anti-oxidant pathway.

The present study exclusively examined the anti-oxidative and anti-aging effects of PKS on *C. elegans* by modulating SKN-1, while the precise upstream and downstream targets within the SKN-1 pathway remain elusive. Studies have shown that the survival time of nematodes can be increased through the mTOR signaling pathway (mTOR), which mainly depends on the transcription factors DAF-16/FOXO and SKN-1/Nrf [43]. However, whether PKS regulates SKN-1 through mTOR signaling pathway deserves further investigation. Several studies had reported that the post transcriptional modiffcation (PTM) of SKN-1 play important roles on regulating *C. elegans* stress resistance [44]. Thus, it is interested in conffrming which type of PTM on SKN-1 was regulated by PKS. Moreover, SKN-1 could regulate mitochondrial function against oxidative stress and contribute to longevity in *C. elegans* [45]. Studies have shown that tomatidine enhances lifespan and healthspan in *C. elegans* through mitophagy induction via the SKN-1/Nrf2 pathway [46]. The excessive accumulation of ROS usually leads to mitochondrial dysfunction and accelerates the process of aging. In this study, PKS-induced oxidative stress resistance is dependent on SKN-1, which suggested mitochondrion may be target organelle for PKS in *C. elegans*. Therefore, the role of PKS in mitochondrion during ageing is worthy of further investigations.

## 5 Conclusion

In summary, PKS exhibit significant in vivo anti-oxidative bioactivity, promoting the nuclear entry of SKN-1, upregulating the expression of relevant anti-oxidant genes, reducing endogenous ROS levels, and enhancing anti-oxidative stress capacity in *C. elegans*. This leads to a slowdown in the aging process, highlighting the potential therapeutic value of PKS in anti-aging applications. However, with the aging of the population, anti-aging has become a hot topic. PKS, as a natural anti-aging component, can provide new ideas for the clinical application of anti-aging therapy. This study explored the impact of PKS on antioxidative and anti-aging effects in *C. elegans*, providing data support for the subsequent exploration, development, and utilization of PKS.

## Funding

The author(s) declare financial support was received for the research, authorship, and/or publication of this article. This work was supported by the 10.13039/501100001809National Natural Science Foundation of China (82074046); the Science and Technology Innovation Program of Hunan Province (2023RC3166); the 10.13039/501100004735Natural Science Foundation of Hunan Province for Excellent Youth Project (2023JJ20033); the Scientific Research Fund of Hunan Provincial Education Department (23A0297); The College Students' innovation and entrepreneurship training program of Hunan Province (S202310541094); The Hunan provincial “Shennong talent” project; Provincial Discipline Construction Project of 10.13039/501100003824Hunan University of Chinese Medicine (Integrated Traditional Chinese and 10.13039/100007159Western Medicine).

## Data availability

Data associated with the study has not been deposited into a publicly available repository. Data are available from the corresponding author on reasonable request.

## CRediT authorship contribution statement

**Yaqi Huang:** Writing – original draft, Formal analysis, Data curation, Conceptualization. **Yetong Wang:** Writing – original draft, Formal analysis, Data curation. **Jia Deng:** Software, Methodology, Data curation. **Sijie Gao:** Methodology, Investigation, Data curation. **Jiakang Qiu:** Formal analysis, Data curation. **Jiawei He:** Software, Methodology, Investigation. **Tong Yang:** Supervision, Methodology. **Nianhua Tan:** Validation, Resources. **Shaowu Cheng:** Writing – review & editing, Funding acquisition. **Zhenyan Song:** Writing – review & editing, Writing – original draft, Visualization, Supervision, Investigation, Funding acquisition, Conceptualization.

## Declaration of competing interest

The authors declare that they have no known competing financial interests or personal relationships that could have appeared to influence the work reported in this paper.
