# Supplementary material for: Research on the anti-oxidant and anti-aging effects of Polygonatum kingianum saponins in Caenorhabditis elegans
Source: Heliyon. 2024 Aug 2;10(15):e35556. doi: 10.1016/j.heliyon.2024.e35556 (PMC11336756; doi:10.1016/j.heliyon.2024.e35556)
Supplement: Multimedia component 1 [file mmc1.docx]

| Primer | Forward primer(5’→3’) | Reverse primer(3’→5’) | Length/bp |
| --- | --- | --- | --- |
| GAPDH | GGAAGTCGCAGCACAAGAT | AGCAGATGGAGCAGAGATGAT | 176 |
| gst-4 | TGATGCTCGTGCTCTTGCT | TGATGCTCGTGCTCTTGCT | 167 |
| gst-7 | CGGATACTTGGTTGGAGACTCT | CGGATACTTGGTTGGAGACTCT | 156 |
| sod-3 | TTGGCTAAGGATGGTGGAGAA | GAACCGCAATAGTGATGTCAGA | 112 |
| hsp16.2 | CGCTATCAATCCAAGGAGAACA | GCAACTGCACCAACATCTACA | 109 |

| PKS/ μg·L^-1^ | time/h | mortality /% |
| --- | --- | --- |
| 0 | 48 | 8 |
|  | 72 | 34 |
| 50 | 48 | 10 |
|  | 72 | 32 |
| 100 | 48 | 8 |
|  | 72 | 32 |
| 200 | 48 | 6 |
|  | 72 | 34 |
| 400 | 48 | 10 |
|  | 72 | 36 |
| 800 | 48 | 8 |
|  | 72 | 34 |
| 1600 | 48 | 10 |
|  | 72 | 34 |
| 3200 | 48 | 8 |
|  | 72 | 36 |

| Number | Diosgenin content/（mg · g^-1^） | Mean/（mg · g^-1^） | RSD% |
| --- | --- | --- | --- |
| 1 | 2.182 | 2.223 | 1.67 |
| 2 | 2.231 |  |  |
| 3 | 2.255 |  |  |
